# Short-term Clinical Results of Manipulation Under Ultrasound-Guided Brachial Plexus Block in Patients with Idiopathic Frozen Shoulder and Diabetic Secondary Frozen Shoulder

**DOI:** 10.2174/1874325001812010099

**Published:** 2018-03-16

**Authors:** Akira Ando, Junichiro Hamada, Yoshihiro Hagiwara, Takuya Sekiguchi, Masashi Koide, Eiji Itoi

**Affiliations:** 1 Department of Orthopaedic Surgery, Matsuda Hospital, 17-1 Sanezawa Aza Tatsutayashiki, Izumiku, Sendai, Japan; 2 Department of Orthopaedic Surgery, Kuwano Kyoritsu Hospital, 2-9-18 Shima, Koriyama, Japan; 3 Department of Orthopaedic Surgery, Tohoku University School of Medicine, 1-1 Seiryomachi, Aobaku, Sendai, Japan

**Keywords:** Manipulation, Shoulder, Ultrasound-guided, Brachial plexus block, Idiopathic frozen shoulder, Diabetic frozen shoulder

## Abstract

**Purpose:**

This study examined the effectiveness of manipulation under ultrasound-guided brachial plexus block in patients with recalcitrant idiopathic frozen shoulder and diabetic secondary frozen shoulder (diabetic frozen shoulder).

**Methods:**

Forty-four idiopathic frozen shoulders and 10 diabetic frozen shoulders with failed conservative treatment for at least 3 months were included in this study. The manipulation was performed under ultrasound-guided brachial plexus block and visual analogue scale, range of motion, and Constant scores were measured before manipulation and at the last follow-up examination.

**Results:**

No major complications were observed during the procedure. Sufficient improvement was not obtained in two patients during the procedure and to avoid complications, the procedure was discontinued and subsequently arthroscopic capsular release was performed. Visual analogue scale, range of motion towards all directions, and Constant scores were significantly improved after the manipulation in both the idiopathic frozen shoulder and diabetic frozen shoulder groups, however the diabetic group showed inferior results compared with those of the idiopathic group.

**Conclusion:**

This manipulation was effective and shortened the duration of symptoms in most of the idiopathic and diabetic frozen shoulders without major complications during the procedure. Diabetic frozen shoulder showed inferior clinical results and difficulty in recovery in range of motion, which indicated that diabetic frozen shoulder should be discussed as a different entity.

## 1 INTRODUCTION

Frozen shoulder is considered to be self-limiting or to recover with or without treatments, but full restoration in range of motion is not always obtained. Although, almost fifty percent of patients with frozen shoulder had some ongoing symptoms even after a few years of conservative treatments, the persistent symptoms are commonly mild [1, 2]. Etiology of this condition is still unclear and subjects of controversy, but various treatment modalities to decrease time to recovery and improve the results of this condition such as oral or intra-articular administration of analgesics or corticosteroids, physical therapy, manipulation under anesthesia, and arthroscopic or open surgery, are reported with controversial results [3-8]. Previous studies emphasized effectiveness of an arthroscopic surgery for the recalcitrant cases as a safe procedure with fewer potential risks of injury to the nerves, tendons, or fracture of the humerus [5, 9, 10]. The therapeutic value of manipulation under general anesthesia was also well reported to shorten the duration of symptoms and recent studies showed that this technique was safer than previously reported when adequate caution was paid for the complications [11-13]. Others claimed that it had little effectiveness and an arthroscopic capsular release should be considered [6, 9, 14, 15]. Recent development of ultrasound technology enabled us to perform brachial plexus or cervical nerve root block safely and effectively, but the effectiveness of manipulation under local anesthesia is little reported [16-18].

Frozen shoulder is divided into primary and secondary due to its etiology. The primary form has an idiopathic pathogenesis, whereas secondary frozen shoulder is diagnosed when it is related to a known cause such as diabetes mellitus [19]. The incidence of frozen shoulder is greater in patients with diabetes mellitus than that in the general population [15]. Poorer results have been reported in diabetic frozen shoulder than idiopathic frozen shoulder concerning conservative treatments, open capsular release, and arthroscopic capsular release [1, 3, 20-22]. Although, remarkable results of manipulation under anesthesia have been reported [11-13, 15], only a few have focused on the diabetic frozen shoulder [15, 20, 23, 24]. Massoud *et al*. reported the successful outcome after the manipulation under general anesthesia in patients with diabetic frozen shoulders [15]. Ogilvie-Harris *et al*. reported poorer results in patients with diabetic frozen shoulder, but detailed results of pain, range of motion, and clinical scores are lacking [20]. The purpose of this study was to examine the effectiveness of the manipulation under brachial plexus block for frozen shoulders with failed conservative treatments and to compare the clinical results between the idiopathic and diabetic frozen shoulders.

## 2 MATERIAL AND METHODS

### 2.1 Subject

A total of 54 consecutive patients, in which conservative treatments failed to improve symptoms, were included in this retrospective study. They consisted of 44 patients with idiopathic frozen shoulder (idiopathic group) and 10 patients with diabetic secondary frozen shoulder (diabetic group). The 10 patients were all non-insulin dependent diabetes mellitus and their average HbA1c was 6.9 ± 2.1 at the initial visit. All patients had routine radiographic evaluation of anteroposterior view in internal and external rotation and outlet view of both shoulders, and patients with radiographic abnormalities such as glenohumeral osteoarthritis, calcifying tendinitis, and superiorly migrated humeral head were excluded. Magnetic resonance imaging was also undertaken and patients with rotator cuff tears were also excluded. Patients with bilateral involvement or traumatic episode were also excluded. The patients were managed conservatively for at least 3 months with oral administration of non-steroidal anti-inflammatory drugs, formal physical therapy, home exercise program, and intra-articular injections of corticosteroid when they were unable to perform the stretching exercises due to pain.

### 2.2 Treatment Technique

The manipulation was performed following a standardized protocol under local anesthesia with the patients in a supine position. Ultrasound-guided interscalene brachial plexus block was performed using 15 ml of 1% mepivacaine hydrochloride in an outpatient setting. The physician lifted the affected extremity and moved the upper arm in flexion and abduction while the scapula was fixed. Next, passive external rotation was performed at 0º of abduction followed by at 90º abduction. Finally, internal rotation at 90º of abduction and horizontal flexion was performed.

### 2.3 Clinical Assessment

The baseline characteristics of the patients such as age, sex, affected side, pretreatment periods, follow-up periods, and existence of diabetes mellitus were examined. History of diabetes mellitus was examined by self-reported questionnaire. Range of motion of the bilateral shoulders, including forward flexion (FF), abduction (ABD), external rotation with the elbow at the side (ER1), and internal rotation (hand behind back, HBB) were measured with the patients in a standing position using caliper before manipulation and at the final visits. Visual analogue scale (VAS) and Constant scores were also measured before manipulation and at the final visits.

### 2.4 Statistical Analysis

Data were expressed as number (percentage), mean (standard deviation), or median (interquartile range). Student t test was used for age, Fisher’s exact test for sex and side, and Mann-Whitney U test was conducted for pretreatment periods, follow-up periods, visual analogue scale, range of motion, and Constant scores. All test were two-sided, and p < 0.05 was accepted as statistical significance. All statistical analyses were performed with SPSS version 23.0 (SPSS Japan Inc., Tokyo, Japan).

## 3 RESULT

No major complications were present during ultrasound-guided brachial plexus block. Sufficient improvement in the range of motion was obtained during the procedure except for the two patients (both idiopathic group). In the two patients, the procedure was discontinued to avoid complications such as fracture and dislocation and subsequently arthroscopic capsular release was performed. The two patients were not included in the following evaluation. Baseline characteristics of both idiopathic and diabetic groups are listed in Table (**1**). There were no significant differences in age, sex, the affected side, pretreatment periods, and follow-up periods between the two groups.

Clinical characteristics of the patients before manipulation and at the final visits are shown in Fig. (**1**). Before manipulation, no significant differences were observed between the two groups as for VAS (p = 0.51), overall range of motion (FF: p = 0.43; ABD: p = 0.34; ER1: p = 0.37), and Constant scores (p = 0.82). Median and interquartile range of HBB before manipulation was at the level of the sacrum (the fifth lumber vertebra, the buttock) and at the sacrum (the fourth lumber vertebra, the buttock) (p = 0.87). At the final visit, all the items in the two groups were improved, but comparing with the idiopathic group, VAS was significantly higher (p = 0.007), motion was restricted (FF: p = 0.002; ABD: p = 0.001; ER1: p = 0.004), and Constant scores were lower (p = 0.002) in the diabetic group. Median and interquartile range of HBB at the final follow up was at the level of the eighth thoracic vertebra (the seventh thoracic vertebra, the ninth thoracic vertebra) and at twelfth thoracic vertebra (eleventh thoracic vertebra, fourth lumber vertebra) (p < 0.001).

## 4 DISCUSSION

Fifty-four patients with recalcitrant idiopathic or diabetic frozen shoulders were treated with the manipulation under ultrasound-guided brachial plexus block. The findings of this study indicated that both idiopathic and diabetic groups were successfully managed with this procedure; however, the diabetic group showed the inferior recovery of VAS, range of motion, and Constant scores compared with the idiopathic group.

Although, the spontaneous course of the frozen shoulder is self-limiting, partial stiffness and pain were reported in 60% of the patients after more than 2 years conservative treatments [1]. An effective and low-risk treatment is needed especially for chronic and painful cases. There has been controversy on the effectiveness of manipulation under anesthesia. Some authors considered manipulation as an effective intervention to be shorten the duration of symptoms [11, 12, 18, 25], whereas others claimed that the procedure had little effectiveness and traumatizing [6, 9, 14]. In this study, most of the patients in both idiopathic and diabetic groups were successfully treated with manipulation under brachial plexus block and recovered in a few months, which indicated that the procedure certainly shortened the periods of symptoms. The reported complications after the manipulation under anesthesia included humeral fractures, rotator cuff tears, glenohumeral dislocations, and injuries to the axillary nerve [17, 25]. Major complications were not noted during the procedure in this study, but a possibility of small injuries remains. Loew *et al*. performed arthroscopy after manipulation under general anesthesia and found 4 patients of iatrogenic superior labrum anterior-posterior lesions and 3 patients of partial tears of the subscapularis tendon in 30 patients [12].

Manipulation under anesthesia was successful in the restoration of function and motion in both the idiopathic and diabetic frozen shoulders, but the restoration was inferior in the diabetic group in this study, which implied that idiopathic frozen shoulder and diabetic frozen shoulder should be understood to be distinct disorders despite the fact that they are both named frozen shoulder. The patients with diabetic etiologies, although with significantly improved range of motion, subjective pain, and Constant scores after the manipulation, showed inferior outcomes in comparison with the idiopathic frozen shoulder group in this study. It is difficult to know the precise mechanism of the inferior prognosis in diabetic patients, but stiffness around the shoulder girdle, that is, stiffness of the muscles (*i.e.* the rotator cuff muscles, pectoralis major, latissimus dorsi, and teres major), fascia, and subcutaneous tissues might affect the outcomes [1, 3, 20-22].

Manipulation under ultrasound-guided brachial plexus block was successfully performed in this study as well as under general anesthesia. Recent development of ultrasound technology enabled us to perform brachial plexus block or cervical nerve root block safely and effectively [16-18]. Sasanuma *et al*. reported that 30 patients with recalcitrant idiopathic frozen shoulders were successfully managed with ultrasound-guided manipulation under cervical nerve root block [17]. This study also indicated that manipulation under brachial plexus block became a treatment option between conservative treatments and arthroscopic capsular release to shorten the time until recovery without major complications.

There are several limitations in this study. Firstly, this study was retrospective and the number of enrolled patients, especially diabetic secondary frozen shoulder, was small. Another criticism is that the mean follow-up period is short. However, long-term follow-up was difficult because most of the patients were satisfied with the outcomes in a few months and they did not hope to visit again.

## CONCLUSION

Manipulation under ultrasound-guided brachial plexus block showed superior clinical results both in the idiopathic and diabetic frozen shoulders. VAS, range of motion to all directions, and Constant scores were significantly improved after the manipulation both in the two groups, however the diabetic group showed inferior results compared with those of the idiopathic group. Diabetic frozen shoulder should be discussed in a different entity.

## Figures and Tables

**Fig. (1):**
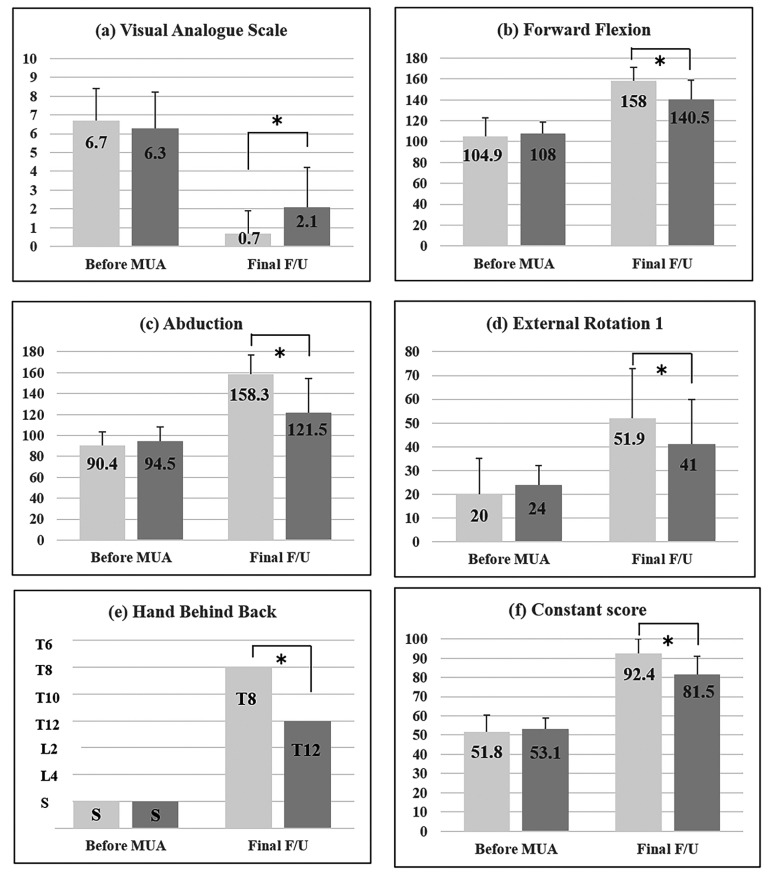
Visual analogue scale (**a**), range of motion (**b-e**), and Constant scores (**f**) before manipulation and at the final follow-up. Light gray bars indicate the idiopathic groups and deep gray bars indicate the diabetic groups, respectively. MUA, manipulation under anesthesia; F/U, follow-up; T, thoracic vertebra; L, lumber vertebra, S, sacrum. * indicates significant difference.

**Table 1:** Baseline characteristics of patients with idiopathic frozen shoulder and diabetic secondary frozen shoulder groups.

	Idiopathic	Diabetic	P value
Shoulders, n	42	10	
Age, years (SD)	58.7 (7.4)	59.4 (7.7)	0.19
Sex, men (%)	14 (33.3)	6 (60.0)	0.16
Affected side, right (%)	20 (47.6)	6 (60.0)	0.3
Pretreatment periods, months (SD)	6.1 (3.7)	6.1 (2.6)	0.68
Follow-up periods, months (SD)	5.1 (2.4)	4.8 (3.5)	0.39

Data were expressed as mean (standard deviation) or number (percentage).
